# Phosphorus

**Published:** 1888-04

**Authors:** A. B. Eadie


### PHOSPHORUS.

Painful feeling of *weakness* and *sensation* of *coldness* (Sec.) or *emptiness* (Cocc.) across the whole abdomen, especially when accompanied by a *sensation* of *heat between scapulæ.*

Shooting in abdomen with *empty feeling;* abdomen *very sensitive, painful* to *touch.*

Small of back *pains as if broken;* a dragging sensation; cannot laugh because of it; cannot move.

Itching pains upward from vagina into pelvis; labor pains distressing but of little use; cutting pains through abdomen; pains in sacrum after labor.

*Exhaustion, fainting,* pale, cold, sudden syncope; lying as if lifeless.

Face *pale,* sunken, or livid; bloated; *dull, glassy, sunken eyes,* with blue rings around them; roaring in ears.

Phosph. is particularly adapted to tall, slender women with dark hair; disposed to stoop; nervous; weak; disposed to sadness; depression, with forebodings of calamity; melancholy; sheds tears or with attacks of involuntary laughter.

Phosph. is aggravated after getting cold; by lying on left side or back, or by light or brilliant objects.

Ameliorations: In the dark; lying on right side; by cold food, or very cold water or ices.

Cold water relieves, but is vomited as soon as it becomes warm.

A. B. Eadie.

